# miR-1250-5p is a novel tumor suppressive intronic miRNA hypermethylated in non-Hodgkin’s lymphoma: novel targets with impact on ERK signaling and cell migration

**DOI:** 10.1186/s12964-021-00707-0

**Published:** 2021-05-27

**Authors:** Min Yue Zhang, Lu Qian Wang, Chor Sang Chim

**Affiliations:** 1 Division of Hematology, Renji Hospital, School of Medicine, Shanghai Jiaotong University, Shanghai, China; 2 Department of Medicine, Queen Mary Hospital, The University of Hong Kong, Pokfulam Road, Pokfulam, Hong Kong

**Keywords:** miR-1250-5p, non-Hodgkin’s lymphoma, DNA methylation, Tumor suppressor miRNA, MAPK1, WDR1

## Abstract

**Background:**

miR-1250 is localised to the second intron of AATK at chromosome 17q25. As a CpG island is present at the putative promoter region of its host gene, AATK, we postulated that the intronic miR-1250-5p is a tumor suppressor miRNA co-regulated with its host gene, AATK, by promoter DNA methylation in non-Hodgkin’s lymphoma (NHL).

**Methods:**

AATK/miR-1250 methylation was studied in healthy controls, including ten normal peripheral blood buffy coats and eleven normal tonsils, ten lymphoma cell lines, and 120 primary lymphoma samples by methylation-specific PCR (MSP). The expression of miR-1250-5p and AATK was investigated by quantitative real-time PCR. Tumor suppressor properties of miR-1250-5p were demonstrated by over-expression of precursor miR-1250-5p in lymphoma cells. The target of miR-1250-5p was verified by luciferase reporter assay.

**Results:**

AATK/miR-1250 methylation was absent in healthy peripheral blood and tonsils, but detected in five (50%) NHL cell lines. AATK/miR-1250 methylation correlated with repression of miR-1250-5p and AATK in NHL cell lines. In completely methylated SU-DHL-6 and SUP-T1 cells, treatment with 5-AzadC led to promoter demethylation and re-expression of both miR-1250-5p and AATK. In primary lymphoma samples, AATK/miR-1250 was frequently methylated in B-cell lymphoma (n = 41, 44.09%) and T-cell lymphoma (n = 9, 33.33%) with a comparable frequency (*P* = 0.318). In SU-DHL-6 and SU-DHL-1 cells, restoration of miR-1250-5p resulted in decreased cellular proliferation by MTS assay, increased cell death by trypan blue staining and enhanced apoptosis by annexin V-PI assay. Moreover, MAPK1 and WDR1 were verified as direct targets of miR-1250-5p by luciferase assay. In 39 primary NHLs, miR-1250-5p expression was shown to be inversely correlated with each of MAPK1 (*P* = 0.05) and WDR1 (*P* = 0.031) by qRT-PCR. Finally, in SU-DHL-1 cells, overexpression of miR-1250-5p led to repression of MAPK1 and WDR1 at both transcript and protein levels, with downregulation of phospho-ERK2 by Western-blotting and inhibition of SDF-1-dependent cell migration by transwell assay.

**Conclusions:**

miR-1250-5p is a novel tumor suppressive intronic miRNA co-regulated and silenced by promoter DNA methylation of its host gene AATK in NHL. MAPK1 and WDR1 are novel miR-1250-5p direct targets rendering inhibition of MAPK/ERK signaling and SDF-1-dependent cell migration, hence implicated in survival and dissemination of lymphoma.

**Video Abstract**

**Supplementary Information:**

The online version contains supplementary material available at 10.1186/s12964-021-00707-0.

## Background

According to origin and lineage of lymphoma cells, non-Hodgkin’s lymphoma (NHL) can be categorized into B-, T- or NK-cell NHL [1]. The most common subtype of NHL is diffuse large B-cell lymphoma (DLBCL), followed by follicular lymphoma (FL), while T-cell lymphoma comprises only 10–15% of all lymphoma [2]. In Hong Kong, the incidence rate of NHL was 8.0/100,000, a rate that have almost doubled in the past two decades (Hong Kong Cancer Registry). The clinical features of NHL are diverse, depending on the subtype of NHL and the site of disease. Patients commonly present painless lymphadenopathy, organomegaly with or without B symptoms (night sweats, weight loss > 10% and fever with temperature > 38 °C) [3].

DNA methylation is an epigenetic modification by which a methyl group (–CH_3_) was enzymatically added to the cytosine at position C5 in CpG dinucleotides without changing DNA sequence, resulting in the formation of 5-methylcytosine [4]. Cancers are characterized by global DNA hypomethylation but gene-specific promoter DNA hypermethylation [5]. Moreover, promoter DNA methylation-mediated silencing of tumor suppressor genes (TSGs), such as p16, SOCS3 and SHP1, has been implicated in lymphomagenesis [6–8].

MicroRNAs (miRNAs) belong to a class of single-stranded non-coding RNAs measuring 19–25 nucleotides [9]. A miRNA targets specific mRNA through sequence-specific binding between miRNA seed region, i.e. the 2nd–7th nucleotides of the miRNA, and seed region binding site (SRBS) on 3′-untranslated region (3′-UTR) of its target gene, resulting in downregulation of the target gene via translational block or mRNA degradation [10, 11]. In carcinogenesis, miRNAs can be tumor suppressive by targeting oncogenes, whereas oncogenic by targeting tumor suppressor genes [12]. Recently, several well-known miRNAs, including oncogenic miR-17-92 and tumor suppressor miR-150, miR-34a, miR-146a, have been implicated in lymphomagenesis [13–16]. In addition, methylation-mediated silencing has been shown to downregulate tumor suppressor miRNAs including miR-155-3p, miR-518a-5p, etc. in NHL [17, 18].

miR-1250 is an intronic miRNA embedded in the second intron of its host gene, apoptosis-associated tyrosine kinase (AATK), localized to 17q25. Recently, miR-1250 was implicated to downregulated in esophageal squamous cell carcinoma and oral cancer tissue compared with normal tissue [19, 20]. Moreover, low expression of miR-1250 was associated with metastatic disease, poorly differentiated histology, and advanced TNM stage in esophageal squamous cell carcinoma [20]. However, the function of miR-1250-5p in carcinogenesis remains unknown. As a CpG island is present at the promoter of miR-1250 host gene AATK, we postulated that miR-1250-5p is a tumor suppressor miRNA silenced by promoter DNA methylation of its host gene AATK in NHL. Herein, promoter DNA methylation of AATK/miR-1250 and its role in lymphomagenesis was investigated based on the discovery of novel miRNA targets, MAPK and WDR1, whereby ERK signaling and SDF1-dependent cell migration were dysregulated.

## Methods

### Patient samples

One hundred and twenty formalin fixed, paraffin-embedded (FFPE) or fresh frozen diagnostic lymph node biopsy tissues including 93 B-cell NHL and 27 T-cell NHL were obtained from five hospitals in Hong Kong (Queen Mary Hospital, Kwong Wah Hospital, Princess Margaret Hospital, United Christian Hospital and Pamela Youde Nethersole Eastern Hospital). Among B-cell NHL cases, there were 56 DLBCL cases, 28 mantle cell lymphoma (MCL) cases, 1 FL cases, 5 Burkitt's lymphoma (BL) cases and 3 small lymphocytic lymphoma (SLL) cases. Among T-cell NHL, there were 20 peripheral T-cell lymphoma (PTCL) and 7 angioimmunoblastic T-cell lymphoma (AITL) cases. The diagnosis of NHL was made according to the WHO (World Health Organization) classification [21]. Eleven FFPE tonsils tissue were collected from healthy individuals undergoing tonsillectomy. Samples were obtained with informed consent. Our study was approved by the Institutional Review Board of Queen Mary Hospital and in accordance with the Declaration of Helsinki.

### Cell culture

Five MCL cell lines (GRANTA-519, JEKO-1, MINO, REC-1, and SP53), two DLBCL cell lines (SU-DHL-6 and SU-DHL-16), two ALK (+) anaplastic large cell lymphoma (ALCL) cell lines (KARPAS-299 and SU-DHL-1) and one T-cell lymphoblastic lymphoma cell line (SUP-T1) were used in this study. REC-1 and SP53 were kind gifts obtained from Prof Raymond Lai (Department of Laboratory Medicine and Pathology, University of Alberta and Cross Cancer Institute). Other cell lines were purchased from Deutsche Sammlung von Mikroogranismen und Zellkulturen (DSMZ) (Braunschweig, Germany). Cell lines were maintained in RPMI-1640 medium (DMEM for GRANTA-519), supplemented with 10–15% fetal bovine serum, 50 U/mL of penicillin and 50 ug/mL streptomycin in a humidified atmosphere of 5% CO_2_ at 37 °C. All cell culture reagents were obtained from Invitrogen (Carlsbad, CA, USA).

### DNA and RNA extraction

DNA Blood Mini kit (Qiagen, Hilden, Germany) was used to extract DNA from NHL cell lines and healthy peripheral blood. Automated DNA extraction system (DNA Tissue Kit from Qiagen) was employed to extract DNA from NHL patient frozen biopsies. DNA isolation from FFPE NHL patient samples and normal tonsils was extracted with QIAamp DNA FFPE Tissue Kit (Qiagen, Hilden, Germany). Extraction of total RNA was performed with Direct-zol™ RNA MiniPrep kit (Zymo Research).

### Methylation-specific polymerase chain reaction (MSP)

**Fig. 1:**
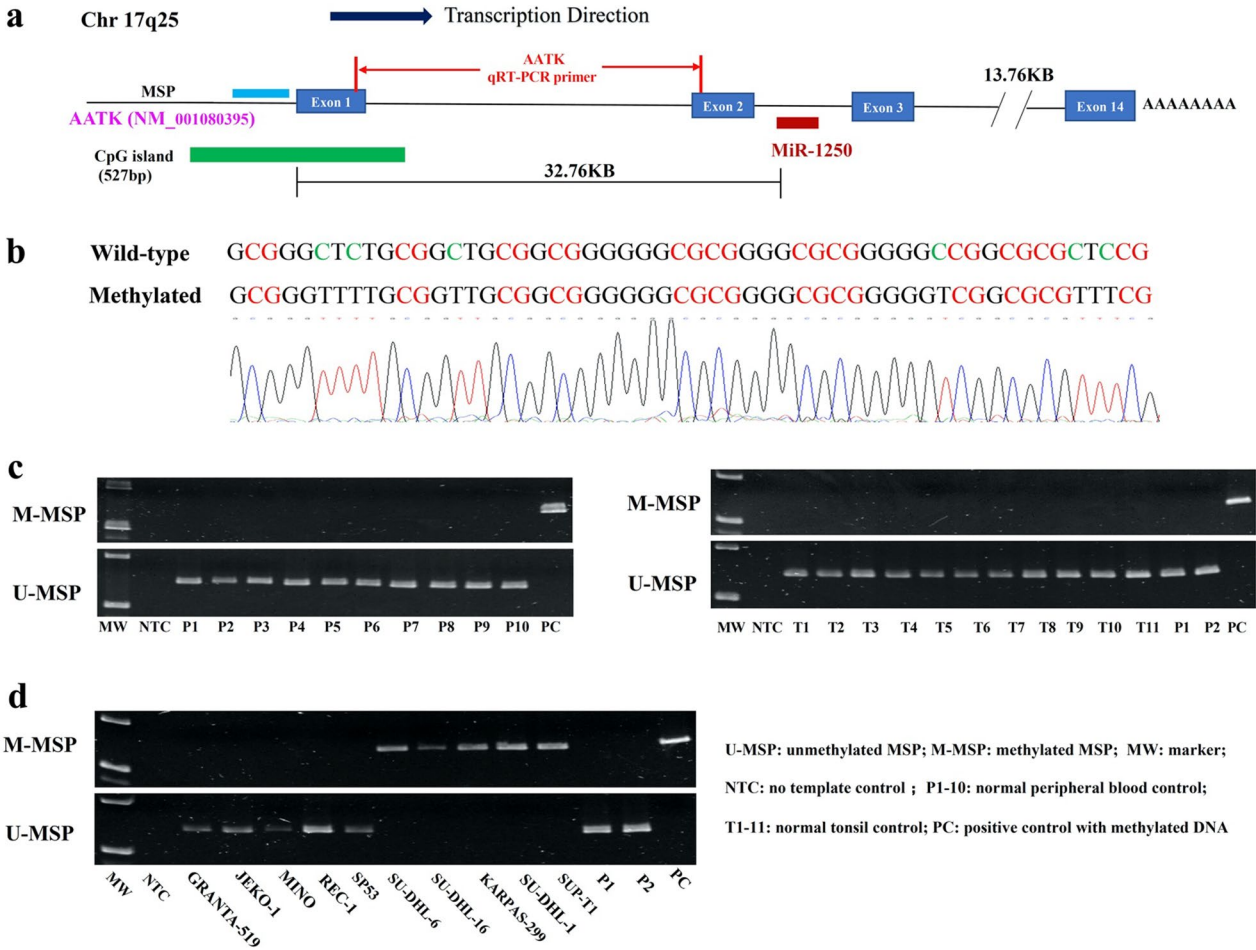
Methylation of AATK/miR-1250 in healthy normal controls and NHL cell lines. **a** Schematic diagram showing the relative locations of miR-1250, AATK gene, CpG island, AATK qRT-PCR primer and amplicons of MSP. **b** Direct sequencing of AATK/miR-1250 M-MSP products from positive control with methylated DNA demonstrated complete bisulfite conversion and MSP specificity. **c** M- and U-MSP showed that AATK/miR-1250 was unmethylated in healthy normal controls, including normal peripheral blood buffy coats (P1–P10) and normal tonsil tissues (T1–T11). **d** In NHL cell lines, M- and U-MSP showed that AATK/miR-1250 was completely unmethylated (UU) in GRANTA-519, JEKO-1, MINO, REC-1 and SP-53, and completely methylated (MM) in SU-DHL-6, SU-DHL-16, KARPAS-299, SU-DHL-1 and SUP-T1

DNA bisulfite treatment was performed for conversion of unmethylated cytosine into uracil with EpiTect Bisulfite Kit (Qiagen, Hilden, Germany). MSP primers were designed at the CpG island embedded at the promoter region of miR-1250 host gene AATK (Fig. 1a). The enzymatically methylated control DNA (Chemicon/Millipore, Billerica, MA, USA) was used as positive control for methylated-MSP (M-MSP) and negative control for unmethylated-MSP (U-MSP). Details of primer sequence and PCR condition for MSP were given in Table 1.

**Table 1:** Primer sequences and PCR reaction conditions for AATK/miR-1250

	Forward primer (5′ to 3′)	Reverse primer (5′ to 3′)	Tm/cycles/MgCl_2_	References
*Methylation-specific PCR (MSP) for AATK/miR-1250*				
M-MSP	TCG GAT TGT ATT AGC GGA GTT TC	CGC CGC AAA TAC GAA ACG	57 °C/35×/2 mM	NA
U-MSP	TTT GGA TTG TAT TAG TGG AGT TTT	ACC ACC ACA AAT ACA AAA CA	55 °C/37×/2 mM	NA
*Quantitative real-time reverse transcription-PCR*				
AATK	CGG GTT CAA GGA GTT TGA GA	GTG AGT GGC AGG ACG TAC AC	NA	NA
WDR1	CAG TGT CTG ACG GTG CAT AA	ACG TCC AGT TTC ACA ACT CC	NA	[23]
GAPDH	ACC ACA GTC CAT GCC ATC ACT	TCC ACC ACC CTG TTG CTG TA	NA	[22]
β-Actin	GGA CTT CGA GCA AGA GAT GG	AGC ACT GTG TTG GCG TAC AG	NA	[24]
*Cloning of luciferase reporter constructs with SRBSs of miR-1250-5p*				
MAPK1	AAG GGC TAG CTT GTG TCC CTG TAT TAC CAA AA	AGG GTC GAC TCT GGG GAA CAT AAC GAA GG	55 °C/35×/2 mM	NA
WDR1	AAG GGC TAG CCC TTC CTT TTC TTT TTC AGT GC	AGG GTC GAC GCG CTC AAA GTG TTT TCA CA	55 °C/40×/2 mM	NA

*M-MSP* methylated MSP, *U-MSP* unmethylated MSP, *Tm* annealing temperature, *SRBS* seed region binding site

### Quantitative real-time reverse transcription-PCR (qRT-PCR)

miR-1250-5p was quantified by TaqMan MicroRNA Reverse Transcription Kit and TaqMan MicroRNA Assay Kit (ABI, Foster City, CA, USA) according to the manufacture’s protocol. RNU48 was used as reference. MAPK1 (Hs01052196_m1, ABI) was quantified by Taqman Gene Expression Assay (ABI, Foster City, CA, USA) and normalized to GAPDH (Cat. Hs00266705_g1). AATK and WDR1 gene were quantified by SYBR Green Master Mix (ABI, Foster City, CA, USA). GAPDH and β-Actin were used as the endogenous control for AATK and WDR1 respectively. 2^−ΔΔCt^ method was used to analyze the expression changes of miR1250-5p and AATK before and after 5-AzadC treatment, and the expression changes of miR-1250-5p, MAPK1 and WDR1 after transfection with precursor miR-1250-5p mimics compared with scrambled oligonucleotides control in SU-DHL-1 cells. Primer sequence for AATK, WDR1, β-Actin and GAPDH were listed in Table 1 [22–24].

### DNA demethylation treatment

SU-DHL-6 and SUP-T1 cells were seeded at a density of 1 × 10^6^ cells/ml in 25 cm^2^ flasks with 0.5–1.5 μM 5-aza-2′-deoxycytidine (5-AzadC) (Sigma–Aldrich, St Louis, MO) for 6 days. 5-AzadC was replaced at every 24 h. Cells were harvested on day 6 for DNA and RNA extraction.

### Transfection of miR-1250-5p

SU-DHL-1 and SU-DHL-6 cells (0.5 × 10^6^ cells/ml) were transfected with either precursor miR-1250-5p mimics or scramble oligonucleotides negative control (Ambion, Austin, TX, USA) at final concentration of 150 nM with Lipofectamine 2000 transfection reagent (Invitrogen, Carlsbad, CA, USA). The transfected cells were cultured under serum-free condition for 48–72 h.

### MTS assay and trypan blue staining

MTS assay was employed to measure cellular proliferation with CellTiter 96® AQ_ueous_ One Solution Cell Proliferation Assay kit (Promega, USA). Transfected cells (2.5 × 10^4^ cells/well) were seeded into a 96-well plate in 100 μl medium. At 48 h post-transfection, 20 μl MTS reagent was added into each well and incubated for 4 h. Then the absorbance reading at 490 nm was recorded. Trypan blue dye exclusion assay was used to determine cellular viability at 48 h after transfection under microscope. Both dead cells and viable cells in five random microscopic fields were counted. Dead cell (%) = (total number of dead cells per microscopic field / total number of viable and dead cells per microscopic field) × 100%. Each assay was repeated in triplicate from three independent transfections.

### FITC annexin-V-PI assay

Cell apoptosis was measured by FITC Annexin-V Apoptosis Detection Kit II (BD Bioscience, USA). Briefly, 1 × 10^5^ cells were harvested, washed with cold PBS and resuspended in 100 μl of Binding Buffer. Then 5 μl of Annexin-V-FITC and 5 μl of PI were added to samples. The cells were incubated for 15 min at room temperature in dark. Lastly, 400 μl of binding buffer were added to each sample. Samples were analyzed by flow cytometry (Beckman Coulter Cytomics FC 500 and NovoCyte Advanteon BVYG). Apoptotic cells included cells in early apoptosis phase (FITC Annexin V positive, PI negative) and late apoptosis phase (FITC Annexin V positive, PI positive). Three independent experiments were performed.

### Transwell migration assay

Cell migration ability was detected by transwell inserts with 8-mm-pore-size in a 24-wells format (Corning, New York, NY, USA). miR-1250-5p mimics or scramble oligonucleotides negative control labelled with Cy3 (Gene Pharma, Shanghai, China) were transfected into SH-DHL-1 cells according to the protocol previously mentioned. At 48 h post-transfection, 1 × 10^6^ transfected cells were resuspended in 100 μl of RPMI 1640 medium containing 2.5% FBS and then seeded into the upper chambers. A total of 600 μl of RPMI 1640 medium with or without SDF-1α (50 ng/ml) was added to the lower chamber. The plate was incubated at 37 °C in 5% CO_2_. After 3 h of incubation, the cells labelled with Cy3 in the lower chamber were counted by Flow Cytometry (NovoCyte Quanteon™) and expressed as number of Cy3-postive cells per 200 μl. The transwell assay were repeated in three independent experiments.

### Western blotting

SU-DHL-1 cells transfected with either precursor miR-1250-5p mimics or scramble negative control were harvested at 72 h post-transfection and lysed in RIPA buffer (Cell Signaling Technology, Danvers, MA, USA) with protease inhibitors and phosphatase inhibitor Cocktail (Cell Signaling Technology). Cell lysates containing 10 μg protein were separated on Mini-PROTEAN TGX™ 10% SDS-PAGE gel (Bio-Rad, Hercules, CA, USA) and transferred to 0.45 μm PVDF membranes (GE Healthcare, Chicago, IL, USA). The membranes were blocked and then incubated with anti-ERK1/2 (1:1000, Cell Signaling Technology), anti-pERK1/2 (1:1000, Cell Signaling Technology), anti-WDR1 (1:1000, Abcam) and anti-actin (1:5000, Cell Signaling Technology) at 4 °C overnight. Then membranes were washed for three times and incubated with Anti-rabbit IgG, HRP-linked Antibody (1:3000, Cell Signaling Technology) for 1 h at room temperature with gently shaking, followed by detection of protein signals with X-ray film.

### Plasmid constructs

The 3′-UTRs of MAPK1 and WDR1 include the putative SRBS of miR-1250-5p (Position: 3934-3941nts of 3′-UTR for MAPK1, 895-901nts of 3′-UTR for WDR1). A 3′-UTR DNA segment of MAPK1 or WDR1 (100–200 bp) containing SRBS of miR-1250-5p was amplified and cloned into the NheI and SalI sites of a dual firefly/renilla luciferase reporter vector, pmirGLO (Promega). MAPK1 or WDR1 3′-UTR mutants with deletion of putative SRBS of miR-1250-5p were synthesized as gBlocks Gene Fragments (Integrated DNA Technologies, Coralville, IA, USA). The sequences of PCR primers and PCR conditions were summarized in Table 1.

### Luciferase reporter assay

In 24-well plate, 0.5 μg wild-type or deletion mutant plasmids and 10 nM precursor miR-1250-5p mimics or scrambled oligonucleotides negative control were co-transfected into HeLa cells (kindly provided by Dr Zou, Department of Medicine, The University of Hong Kong) using Lipofectamine 2000 transfection reagent. At 48 h post-transfection, the luminescent signal was quantified by Dual-Luciferase Reporter Assay System (Promega) by CLARIOstar (BMG Labtech). Firefly luciferase activity was normalized by Renilla luciferase activity. Each experiment was conducted in triplicate from three independent transfections.

### Statistical analysis

The mean expression of miR-1250-5p or AATK between methylated and unmethylated NHL cell lines were compared by Student’s t-test. The differences of trypan blue exclusion assay, MTS assay, FITC Annexin-V-PI assay and transwell migration assay between NHL cells transfected with miR-1250-5p mimics and scrambled oligonucleotides control were compared by Student’s t-test. The difference of AATK/miR-1250 methylation frequency in different subtypes of NHL primary samples was analyzed by χ^2^ test. All *P* values were 2-sided. *P* < 0.05 was considered as significant difference.

## Results

### Promoter DNA methylation of AATK/miR-1250 in normal healthy controls and NHL cell lines

DNA methylation of AATK/miR-1250 promoter was examined in bisulfite-converted DNA of normal healthy controls, comprising peripheral blood buffy coats (n = 10) and normal tonsils (n = 11), and NHL cell lines (n = 10) by MSP. Direct sequencing analysis of the M-MSP products from positive control with methylated DNA demonstrated complete conversion of unmethylated but not methylated cytosine residues into thymidine residues after PCR, confirming complete bisulfite conversion and specificity of MSP (Fig. 1b). Moreover, by MSP, AATK/miR-1250 was unmethylated in all normal healthy controls (Fig. 1c). In NHL cell lines, AATK/miR-1250 was completely unmethylated (UU) in GRANTA-519, MINO, SP-53, REC-1 and JEKO-1, but completely methylated (MM) in SU-DHL-6, SU-DHL-16, KARPAS-299, SU-DHL-1 and SUP-T1 (Fig. 1d). Taken together, these data indicated that methylation of AATK/miR-1250 was tumor-specific in NHL.

### Promoter DNA methylation and expression of AATK and its intronic miR-1250-5p in NHL cell lines

**Fig. 2:**
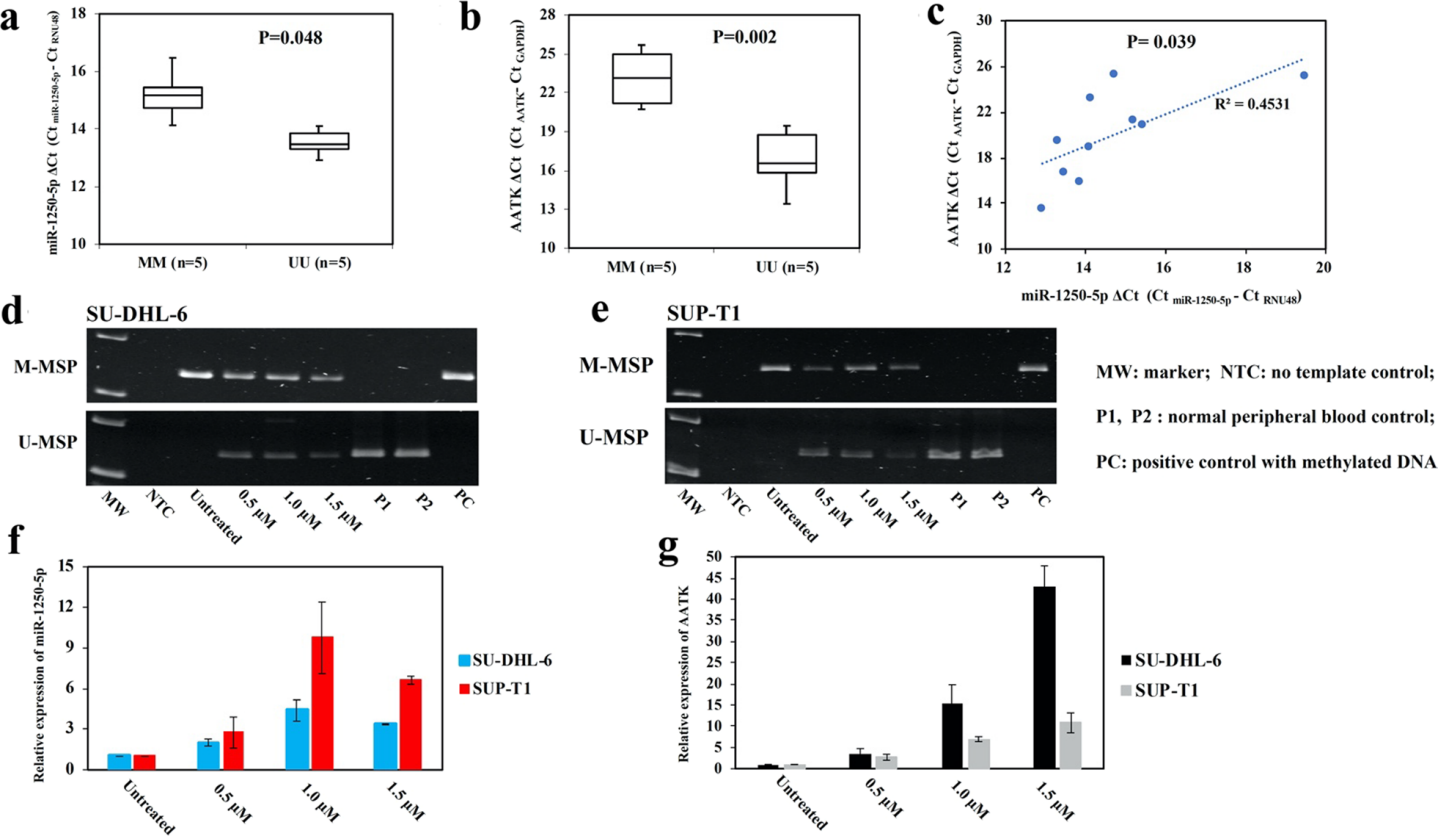
Methylation and expression of AATK/miR-1250 in NHL cell lines. **a** and **b** By qRT-PCR, AATK/miR-1250 methylation measured by MSP was associated with lower expression of miR-1250-5p (**a**) and its host gene AATK (**b**). **c** By plotting the expression of AATK against miR-1250-5p, a concordant expression between miR-1250-5p and its host gene AATK was demonstrated. (**d**–**g**) In SU-DHL-6 and SUP-T1 cells completely methylated for AATK/miR-1250, treatment with 5-AzadC for 6 days led to AATK/miR-1250 promoter demethylation by MSP (**d**, **e**), and associated with re-expression of both intronic miR-1250-5p (**f**) and its host gene AATK by qRT-PCR (**g**). Columns represented mean ± 1SD from three experiments in triplicate

To study whether AATK/miR-1250 methylation was associated with expr**e**ssion of AATK and its intronic miR-1250-5p, expression of miR-1250 and AATK were assessed by qRT-PCR, and correlated with MSP data in NHL cell lines. Data confirmed that the mean expression of both miR-1250-5p and its host gene AATK was significantly lower in completely methylated cell lines than that in completely unmethylated cell lines (miR-1250-5p: *P* = 0.048, Fig. 2a; AATK: *P* = 0.002, Fig. 2b). Moreover, co-expression between AATK and its intronic miR-1250-5p was demonstrated by a positive slope generated from plotting the expression of AATK against miR-1250-5p (R^2^ = 0.4531, *P* = 0.033, Fig. 2c).

Furthermore, to verify if AATK and its intronic miR-1250-5p were co-regulated by reversible methylation-mediated silencing, SU-DHL-6 and SUP-T1 cells completely methylated for AATK/miR-1250 were treated with a hypomethylating agent, 5-AzadC. 5-AzadC treatment resulted in promoter demethylation of AATK/miR-1250 as evidenced by the emergence of U-MSP signal on day 6 (Fig. 2d, e), and concomitant re-expression of both miR-1250-5p (Fig. 2f) and its host gene AATK (Fig. 2g). Therefore, these data indicated that AATK and its intronic miR-1250-5p were reversibly silenced by promoter DNA methylation in NHL cells.

### Promoter DNA methylation of AATK/miR-1250 in primary samples of NHL

**Fig. 3:**
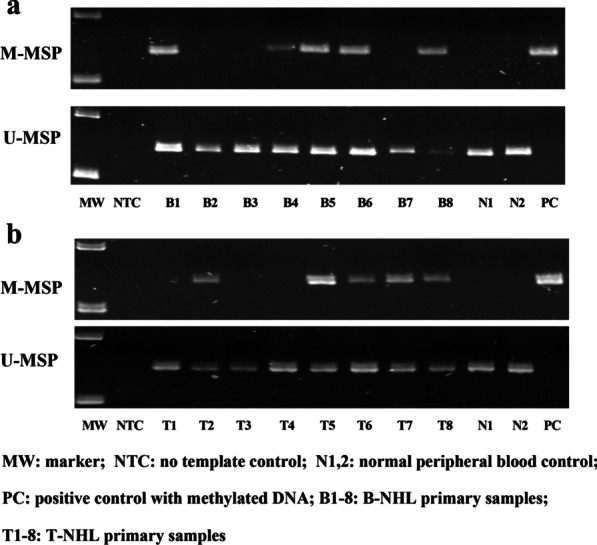
Methylation of AATK/miR-1250 in NHL primary samples. **a** and **b** Representative M- and U-MSP illustrated methylation of AATK/miR-1250 in primary samples of B-cell NHL (**a**) and T-cell NHL (**b**)

MSP was performed using bisulfite-converted DNA of primary NHL samples comprising 93 B-cell NHL and 27 T-cell NHL. AATK/miR-1250 methylation was detected in 41 (44.09%) B-cell NHL and 9 (33.33%) T-cell NHL cases (Table 2; Fig. 3a, b), hence AATK/miR-1250 were frequently hypermethylated in both B-cell and T-cell NHL with a comparable frequency (*P* = 0.318).

**Table 2:** AATK/miR-1250 methylation frequency in NHL primary samples

Disease subtype	Sample size	Methylation (%)
B-cell NHLs	93	41 (44.09%)
Diffuse large B-cell lymphoma (DLBCL)	56	35 (62.50%)
Mantle cell lymphoma (MCL)	28	2 (7.14%)
Follicular lymphoma (FL)	1	0 (0%)
Burkitt’s lymphoma (BL)	5	4 (80.00%)
Small lymphocytic lymphoma (SLL)	3	0 (0%)
T-cell NHLs	27	9 (33.33%)
Peripheral T-cell lymphoma (PTCL)	20	8 (40.00%)
Angioimmunoblastic T-cell lymphoma (AITL)	7	1 (14.29%)

### Effect of miR-1250-5p overexpression in NHL cells

**Fig. 4:**
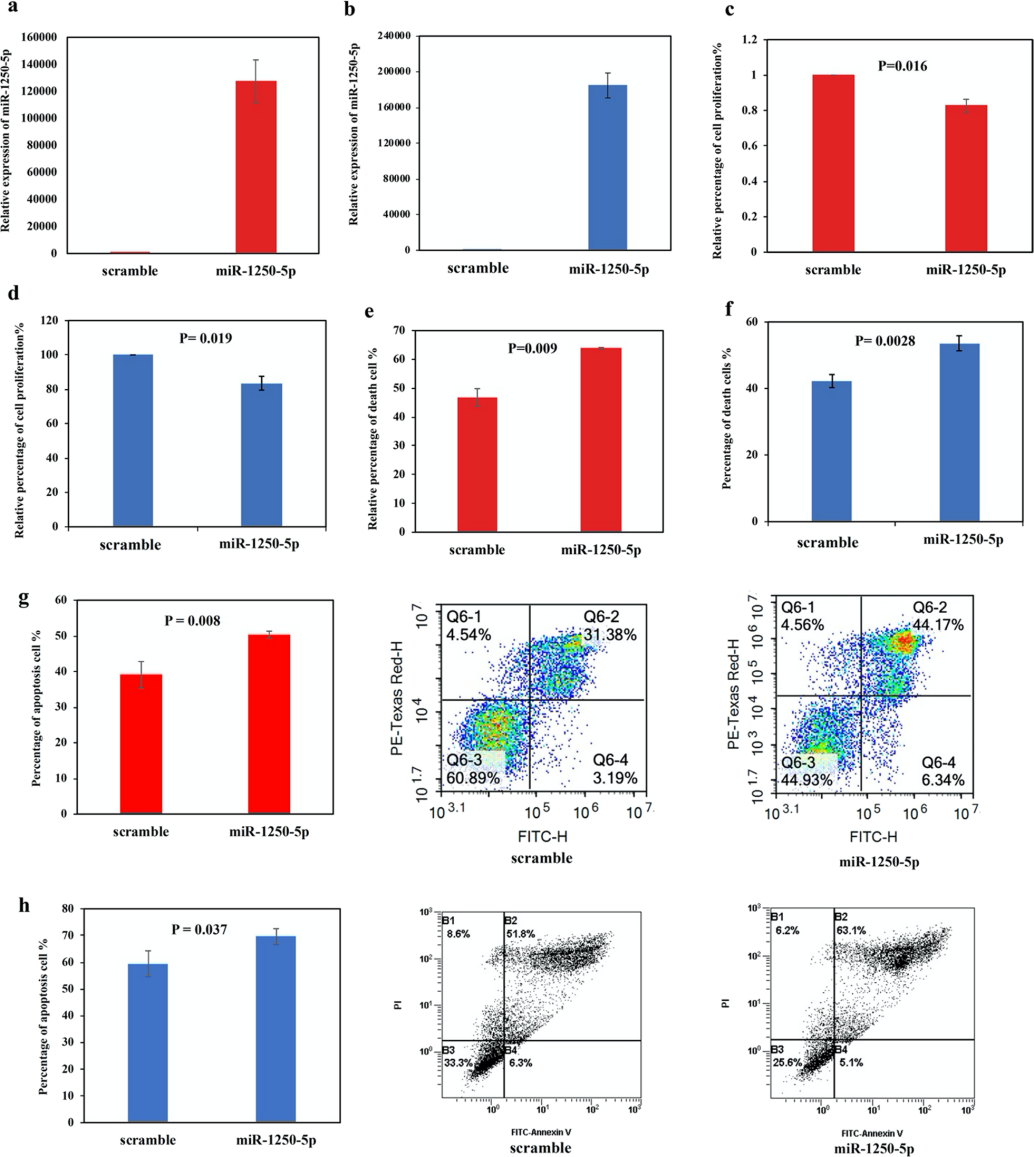
Effect of miR-1250-5p overexpression in NHL cells. **a** and **b** In SU-DHL-6 (**a**) and SU-DHL-1 (**b**) cells, miR-1250-5p mimics were transfected using Lipofectamine 2000, followed by qRT-PCR analysis of miR-1250-5p expression at 48 h after transfection as compared with scramble negative control. **c** and **d** Cellular proliferation upon overexpression of miR-1250-5p was analyzed by MTS assay in SU-DHL-6 (**c**) and SU-DHL-1 (**d**) cells. **e** and **f** Number of dead cells upon overexpression of miR-1250-5p was calculated by trypan blue exclusion assay in SU-DHL-6 (**e**) and SU-DHL-1 (**f**) cells. **g** and **h** Apoptosis upon overexpression of miR-1250-5p was analyzed by FITC Annexin-V-PI assay in SU-DHL-6 (**g**) and SU-DHL-1 (**h**) cells. Columns represented mean ± 1SD from three independent experiments

As AATK/miR-1250 was frequently methylated in NHL primary samples, the tumor suppressor function of miR-1250-5p was studied by overexpression of miR-1250-5p in completely methylated NHL cells, including B-cell lymphoma SU-DHL-6 cells and T-cell lymphoma SU-DHL-1 cells. By qRT-PCR, miR-1250-5p was overexpressed in SU-DHL-6 and SU-DHL-1 cells at 48 h post-transfection (Fig. 4a, b). Compared with scramble negative control, overexpression of miR-1250-5p led to reduced cellular proliferation by MTS assay (SU-DHL-6: *P* = 0.016, Fig. 4c; SU-DHL-1: *P* = 0.019 Fig. 4d), increased cell death by trypan blue exclusion assay (SU-DHL-6: *P* = 0.009, Fig. 4e; SU-DHL-1: *P* = 0.0028 Fig. 4f), and enhanced apoptosis by FITC Annexin V-PI analysis (SU-DHL-6: *P* = 0.008, Fig. 4g; SU-DHL-1: *P* = 0.037 Fig. 4h). Taken together, our data testified the tumor suppressor role of miR-1250-5p in NHL cells.

### Identification of MAPK1 and WDR1 as novel direct targets of miR-1250-5p in NHL cells

**Fig. 5:**
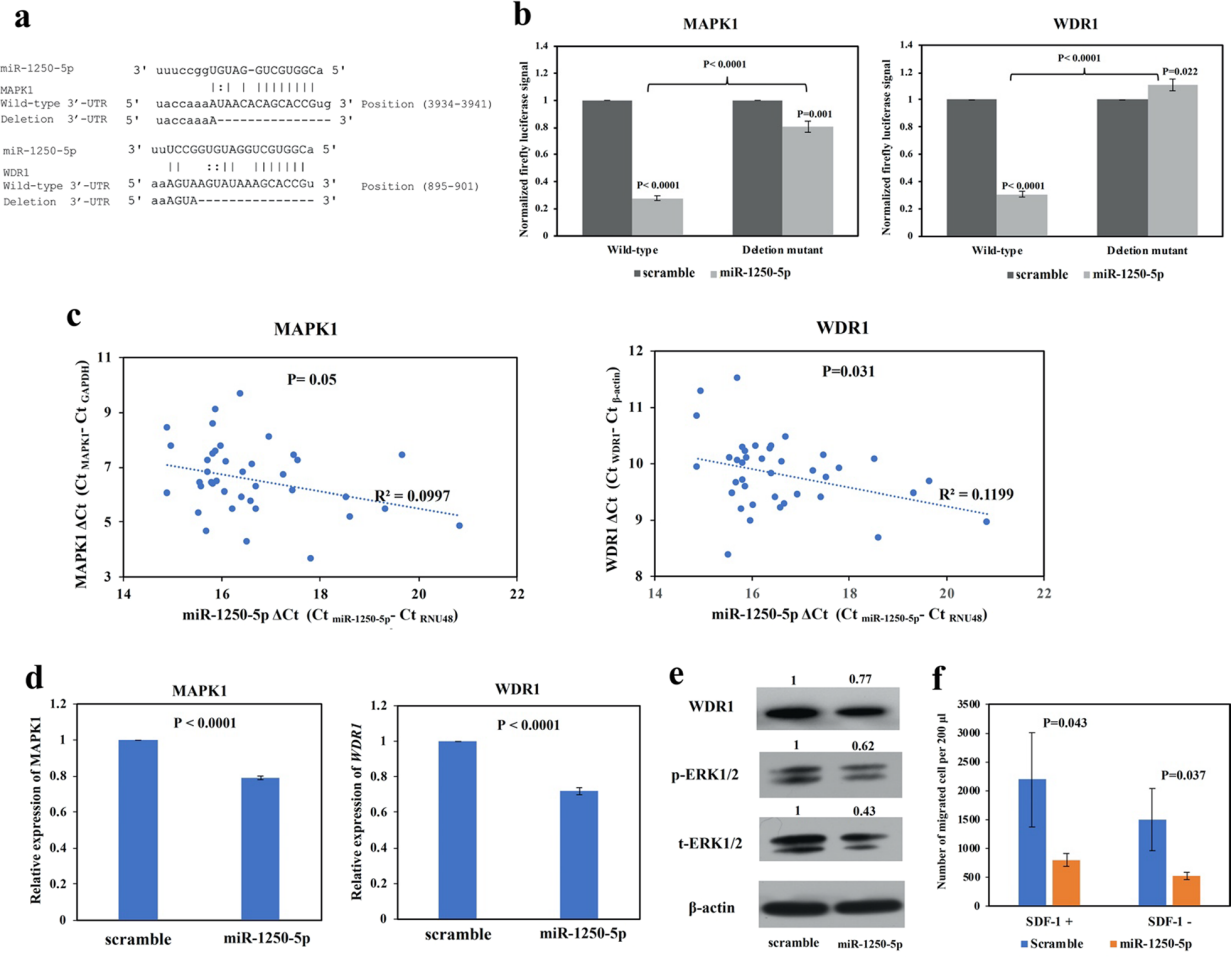
Identification of MAPK1 and WDR1 as novel direct targets for miR-1250-5p. **a** Bioinformatics analyses showed the putative sequence-specific binding between seed region of miR-1250-5p and SRBS on the 3′UTR of MAPK1 and WDR1. The deletion mutant was generated according to the bottom line of each binding site. **b** By luciferase assay, in HeLa cells, the luciferase activity of plasmids containing wild-type 3′-UTR of MAPK1 or WDR1 were inhibited upon co-transfection with miR-1250-5p mimics, as compared with scramble negative control. In contrast, the luciferase activity of plasmids containing deletion mutant, as shown in (**a**), was significantly restored. **c** In NHL primary samples, using ΔCt, the expression of MAPK1 and WDR1 was plotted against miR-1250-5p, and significant inverse correlations were shown. **d** By qRT-PCR, the expression of each of MAPK1 and WDR1 was downregulated in SU-DHL-1 cells transfected with miR-1250-5p mimics, as compared with scramble negative control. **e** Western blotting showed that, upon overexpression of miR-1250-5p, WDR1, phospho-ERK1/2 and total-ERK1/2 were downregulated in SU-DHL-1 cells. β-actin was set as endogenous control. **f** Cell migration upon transfection of miR-1250-5p mimics and scramble negative control in SU-DHL-1 cells in the presence or absence of SDF-1 was analyzed by transwell migration assay. Columns represented mean ± 1SD from three independent experiments

To investigate the mechanisms of the tumor suppressor effect of miR-1250-5p, putative targets of miR-1250-5p were predicted using bioinformatics platform miRwalk 2.0, comprising 7 common miRNA target prediction databases, including miRWalk, Microt4, miRanda, miRDB, RNA22, Targetscan, Pictar2 [25]. The selection criteria included: (1) the same target was predicted by more than four databases; (2) matching to the 7mer-A1, 7mer-M8 and 8-mer miRNA seed sequences. This algorithm yielded a total of 230 potential targets (Additional file 1: Table S1). Next, we selected the targets which were oncogenic or overexpressed in cancer for further study. Two novel targets including Mitogen-Activated Protein Kinase 1 (MAPK1, also known as ERK2), a downstream effector for MAPK/ERK signaling pathway [26], and WD Repeat Domain 1 (WDR1, also known as Aip1), implicated in cancer cell migration and progression [27], were identified as putative targets of miR-1250-5p for further study (Fig. 5a).

Firstly, whether MAPK1 and WDR1 were direct targets of miR-1250-5p was confirmed by luciferase reporter assay. Luciferase reporter constructs containing wild-type or deletion mutant 3′-UTR of MAPK1 and WDR1 were generated (Fig. 5a). For each of MAPK1 and WDR1, luciferase vector containing the wild-type or deletion mutant 3′-UTR was co-transfected with miR-1250-5p mimics or scramble negative control into HeLa cells. At 48 h post-transfection, as compared with scramble negative control, overexpression of miR-1250-5p significantly inhibited luciferase activity for the luciferase constructs containing wild-type 3′-UTR of MAPK1. However, the inhibition was significantly restored when co-transfection of miR-1250-5p with the deletion mutant 3′-UTR of MAPK1, in which SRBS of miR-1250-5p was deleted (Fig. 5b). Similarly, as compared with scramble negative control, overexpression of miR-1250-5p significantly suppressed luciferase activity for the luciferase constructs with wild-type 3′-UTR of WDR1. Conversely, the suppression was completely restored when co-transfection of miR-1250-5p with the deletion mutant 3′-UTR of WDR1, in which miR-1250-5p SRBS was deleted (Fig. 5b). Taken together, our data suggested that MAPK1 and WDR1 were novel direct targets of miR-1250-5p.

Secondly, qRT-PCR of miR-1250-5p, MAPK1 and WDR1 were performed in sub-cohort of 39 NHL primary samples including 2 MCL cases, 18 DLBCL cases, 1 FL case, 5 BL cases, 3 SLL cases, 3 PTCL cases and 7 AITL cases. Among these 39 NHL samples, 15 cases were partially methylated for AATK/miR-1250, 24 cases were completely unmethylated for AATK/miR-1250. The results demonstrated that the expression of miR-1250-5p was inversely correlated with that of MAPK1 (*P* = 0.05, R^2^ = 0.0997, Fig. 5c) and WDR1 (*P* = 0.031, R^2^ = 0.1199, Fig. 5c), consistent with repression of MAPK1 and WDR1 by miR-1250-5p in primary NHL samples.

Finally, in SU-DHL-1 cells, overexpression of miR-1250-5p resulted in significant downregulation of MAPK1 (total-ERK2) and WDR1 at both transcript level by qRT-PCR (Fig. 5d) and protein level by Western blotting (Fig. 5e). Functionally, by Western blotting, restoration of miR-1250-5p led to downregulation of phospho-ERK2, the active form of MAPK1 in MAPK/ERK signaling (Fig. 5E). As WDR1 has been shown to inhibit SDF-1-dependent migration of lymphoma cells [28], cell migration was studied by transwell assay in SU-DHL-1 cells upon overexpression of miR-1250-5p. Overexpression of miR-1250-5p significantly decreased SH-DHL-1 cell migration as compared with scramble negative control in the presence of SDF-1 (Fig. 5f). Moreover, migration of SH-DHL-1 cells transfected with miR-1250-5p mimics or scramble oligonucleotides negative control were decreased in the absence of SDF-1 (Fig. 5f), consistent with SDF-1-mediated cell migration. Collectively, these data suggested the inhibitory role of miR-1250-5p on MAPK/ERK signaling pathway and SDF-1-dependent cell migration via direct targeting of MAPK1 and WDR1 respectively.

## Discussion

In this study, firstly, we have demonstrated that AATK/miR-1250 was frequently methylated in NHL cell lines and NHL primary samples, but unmethylated in healthy normal controls, hence a tumor-specific pattern of methylation in NHL, similar to tumor-specific pattern of methylation of other tumor suppressor miRNAs, such as miR-155-3p [18], miR-124-1 [29], and miR-203 [30] in NHL cells. Conversely, there are miRNAs, such as miR-373 and miR-517c [31], methylated in both tumor cells and their normal counterparts, and hence a tissue-specific but not tumor-specific pattern of methylation, hence unlikely important in carcinogenesis.

Secondly, our study illustrated that the intronic miR-1250-5p was co-regulated by promoter DNA methylation of its host gene AATK in NHL, similar to other intronic miRNAs, such as miR-342-3p [32] and miR-28-5p [33], which were shown to be co-regulated by promoter DNA methylation of their host genes, EVL and LPP, respectively, in multiple myeloma. Therefore, our result supported the view that intronic miRNA is co-expressed and co-regulated by promoter DNA methylation with its host gene [34]. Notably, another two intronic miRNAs, miR-338 and miR-657, are embedded in the 7th intron of AATK. Whether intronic miR-338 and miR-657 are also co-regulated and co-expressed with host gene AATK by promoter DNA methylation in NHL cells remains unknown and warrants future study.

Thirdly, in primary samples, AATK/miR-1250 methylation was frequently detected in both B-cell and T-cell NHL. Moreover, unlike miR-124-1 [29] and miR-129-2 [35], which were preferentially methylated in B-cell rather than T-cell NHL, AATK/miR-1250 methylation was comparable between B-cell and T-cell NHL.

Fourthly, while functional study of miR-1250-5p is scanty, our data showed for the first time that miR-1250-5p was a tumor suppressor miRNA in NHL, as evidenced by inhibition of cellular proliferation and increase of apoptotic cell death upon overexpression of miR-1250-5p in both B-cell and T-cell lymphoma cells. Moreover, by luciferase assay, MAPK1 and WDR1 were confirmed to be novel direct targets of miR-1250-5p, and hence the tumor suppressive role of miR-1250-5p was mediated by direct targeting of MAPK1 and WDR1. On the other hand, functional activation of MAPK/ERK signaling pathway can be demonstrated by phosphorylation of both MAPK2 (ERK1)/MAPK1 (ERK2) [26]. In cancers including NHL, aberrant activation of MAPK/ERK signaling pathway has been shown to enhance cell growth, invasion and metastasis but inhibit apoptosis [26, 36–38]. In this connection, our data suggested that epigenetic silencing of miR-1250-5p could result in enhancing the expression of total and phosphorylated MAPK1(ERK2), contributing to activation of MAPK/ERK signaling pathway. Similarly, miR-422a was shown to inhibit MAPK/ERK signaling by direct targeting and hence downregulation of MAPK1(ERK2) [39]. Conversely, other than regulating activation of MAPK/ERK signaling pathway by altering expression of MAPK2(ERK1)/MAPK1(ERK2), miR-3151 was shown to inhibit MAPK/ERK signaling activity by direct targeting of MADD, which is crucial in facilitating phosphorylation and hence activation of ERK1/2 protein, and hence resulting in reduced phosphorylation, but not expression, of ERK1/2 [40].

On the other hand, as confirmed by luciferase reporter assay, our data also suggested that methylation-mediated silencing of miR-1250-5p could result in upregulation of another direct target, WDR1. WDR1 was found to be upregulated and associated with poor prognosis in breast and lung cancer [41–43]. In lung cancer cells, the oncogenic function of WDR1 in the regulation of cellular proliferation was shown to be mediated by dephosphorylation and hence increased nuclear translocation of YAP protein, a downstream effector for Hippo signaling pathway, hence activation of the transcription of pro-proliferative genes, such as cyclin A2, cyclin B1, cyclin D1, cyclin E and Cdk1 [41]. Moreover, in breast cancer cells, knockdown of WDR1 resulted in inhibition of cancer cell migration and invasion [42, 43]. By contrast, in the absence of SDF-1, the inhibitory effect of cell migration by knockdown of WDR1 was weakened, confirming the oncogenic property of WDR1 in the promotion of SDF-1-dependent migration of lymphoma cell [28]. Similarly, our data demonstrated that overexpression of miR-1250-5p led to repression of WDR1 and suppression of SDF-1-dependent migration in NHL cell.

## Conclusions

To sum up, miR-1250-5p is a tumor suppressor intronic miRNA co-regulated and silenced by promoter DNA methylation of its host gene AATK in a tumor-specific and reversible manner in NHL. Moreover, MAPK1 and WDR1 are novel targets of miR-1250-5p, regulating MAPK/ERK signaling and SDF-1-dependent cell migration, hence implicated in cell survival and lymphoma dissemination. Finally, in primary NHL, AATK/miR-1250 methylation was frequently detected in both B- and T-cell NHL, hence implicated in lymphomagenesis.

## Supplementary Information

**Additional file 1: Table S1.** List of 230 potential targets of miR-1250-5p predicted by bioinformatics platform miRwalk 2.0.

## Data Availability

The datasets generated for this study are available on request to the corresponding author.

## Abbreviations

NHL: non-Hodgkin’s lymphoma
DLBCL: diffuse large B-cell lymphoma
FL: follicular lymphoma
TSG: tumor suppressor gene
miRNA: microRNA
SRBS: seed region binding site
3′-UTR: 3′-untranslated region
AATK: apoptosis-associated tyrosine kinase
MCL: mantle cell lymphoma
PTCL: peripheral T-cell lymphoma
AITL: angioimmunoblastic T-cell lymphoma
BL: Burkitt's lymphoma
SLL: small lymphocytic lymphoma
FFPE: formalin fixed, paraffin-embedded
ALCL: anaplastic large cell lymphoma
MSP: methylation-specific polymerase chain reaction
M-MSP: methylated-MSP
U-MSP: unmethylated-MSP
qRT-PCR: quantitative real-time reverse transcription-PCR
5-AzadC: 5-Aza-2′-deoxycytidine
MAPK1: mitogen-activated protein kinase 1
WDR1: WD repeat domain 1
